# Determining passenger car equivalent (PCEs) for pretimed signalized intersections with severe motorcycle composition using Bayesian linear regression

**DOI:** 10.1371/journal.pone.0256620

**Published:** 2021-09-02

**Authors:** Sugiarto Sugiarto, Fadhlullah Apriandy, Yusria Darma, Sofyan M. Saleh, Muhammad Rusdi, Tomio Miwa

**Affiliations:** 1 Civil Engineering Department, Universitas Syiah Kuala, Banda Aceh, Indonesia; 2 Remote Sensing and Cartography Laboratory, Universitas Syiah Kuala, Banda Aceh, Indonesia; 3 Institute of Materials and Systems for Sustainability, Nagoya University, Nagoya, Japan; University of Shanghai for Science and Technology, CHINA

## Abstract

Pretimed signalized intersection is known as a common source of congestion, especially in urban heterogeneous traffic. Furthermore, the accuracy of saturation flow rate is found to cause efficient and vital capacity estimation, in order to ensure optimal design and operation of the signal timings. Presently, the traffic also consists of diverse vehicle presence, each with its own static and dynamic characteristics. The passenger car equivalent (PCE) in an essential unit is also used to measure heterogenous traffic into the PCU (Passenger Car Unit). Based on the collection of observed data at three targets in Banda Aceh City, this study aims to redetermine the PCEs by using Bayesian linear regression, through the Random-walk Metropolis-Hastings and Gibbs sampling. The result showed that the obtained PCE values were 0.24, 1.0, and 0.80 for motorcycle (MC), passenger car (PC), and motorized rickshaw (MR), respectively. It also showed that a significant deviation was found between new and IHCM PCEs, as the source of error was partially due to the vehicle compositions. The present traffic characteristics were also substantially different from the prevailing conditions of IHCM 1997. Therefore, the proposed PCEs enhanced the accuracy of base saturation flow prediction, provided support for traffic operation design, alleviated congestion, and reduced delay within the city, which in turn improved the estimation of signalized intersection capacity.

## Introduction

Pretimed signalized intersections are known as the major types of traffic facilities that significantly influences the infrastructure of road networks, which are used for the efficient operation of urban corridors. However, an inefficient design capacity is found to cause congestion and delay in the road network. Previous studies showed that congestion within a city center provided transport externalities, such as long travel times, high vehicle operating cost, air pollution, excessive energy consumption, and serious economic loss [1–3]. They also concluded that the quality of service facilities at arterial road segments with U-turn sections and on-street parking, were the major source of traffic congestion in Banda Aceh, Indonesia. Furthermore, the main cause of traffic bottleneck is the poor trade-off between demand and infrastructural supply [4,5]. Therefore, understanding the traffic mechanism at a signalized intersection is important for alleviating congestion. Two strategies were also applied in order to deal with traffic breakdown, which helped in improving the capacity with a unique and refined design [6], as well as decreasing delay with optimized signal control [7,8].

According to the condition of heterogeneous traffic, movements and compositions are generally characterized by the presence of diverse vehicle types, each with static and dynamic characteristics. This includes light and heavy vehicles, motorcycles, and rickshaws. The movements and compositions also have different effects on the traffic characteristics. The Indonesian Highway Capacity Manual [9], is known to guide engineers and planners in planning, designing, and operating traffic facilities, which includes pretimed signalized intersections. This indicates that the accurate estimation of the signalized intersection capacity plays an essential role in designing and operating a facility. These intersections are known to be widely operated in Banda Aceh, which is the capital of Aceh province, Indonesia. This type of facility is also a common source of congestion and delay in the road network, due to the heterogeneous compositions and non-lane based movements of traffic [10]. Furthermore, saturation flow rate is an essential task to evaluate the signal timing and performance of the signalized intersection, which is found to substantially correspond with the passenger equivalent unit (PCE). This indicates that the PCEs are fundamental input parameters used for saturation flow rate and capacity calculation, especially pretimed signal timing design.

Two approaches are often used in estimating saturation flow rates, namely (1) field measurement (direct method), (2) existing formulae from traffic codes, e.g., IHCM and USHCM in Indonesia and US, respectively. According to IHCM, the saturation flow rate is obtained by multiplying the BSFR (base saturation flow rate) with adjustment factors, such as the city size, side friction, grade, parking distance from the stop line, as well as proportion of right and left-turning vehicles.

The accuracy of capacity estimation is also vital to ensuring optimal signal timings, minimizing delay, and mitigating congestion. This study proposes a calibration for PCE values, by considering the interactive effects of the vehicle compositions, especially for pretimed signal timing. Based on the use of primary and observed data collected at three pretimed signalized intersections in Banda Aceh, this study aims to model and determine the PCE values, by adopting the use of multiple linear regression method with synchronous data counting [11,12]. This is further carried out by using the Bayesian technique, in order to calibrate PCE values in Banda Aceh city. The remainder of this study is organized as, Sections 2, 3, and 4, which reviews the existing related studies and describes the data collection, model formulation, and obtained estimation results, as well as present the discussions and conclusions.

## Methods

### PCE and model estimation

The IHCM used was proposed by the Directorate General Highways Ministry of Public Works, in 1997. This tool is essential in planning, designing, and operating traffic facilities in Indonesia. The IHCM manual was also designed to allow engineers and planners predict the quality of facility services, under a provided set of traffic, geometry, and environmental conditions. Based on this tool, the SFR (saturation flow rate) is defined as the product between the base saturation flow rate (S_0_) and correction factors (F), which are used in considering the deviation of actual conditions from a set of pre-determined (ideal) situations, as shown in Eqs 1 and 2.

$$\text{S}={\text{S}}_{0}{\text{F}}_{\text{CS}}{\text{F}}_{\text{sf}}{\text{F}}_{\text{g}}{\text{F}}_{\text{p}}{\text{F}}_{\text{rt}}{\text{F}}_{\text{lt}}$$ (1)

$$\text{S}=600{\text{W}}_{e}{\text{F}}_{\text{CS}}{\text{F}}_{\text{sf}}{\text{F}}_{\text{g}}{\text{F}}_{\text{p}}{\text{F}}_{\text{rt}}{\text{F}}_{\text{lt}}$$ (2)

where,

S = the saturation flow rate (PCU/h),
S_0_ = the base saturation flow rate (PCU/h),
F-terms = the adjustment factors for the city size, side friction, grade, as well as proportion of right and left-turning vehicles, respectively.

There were also continuous efforts working on the modelling and evaluation of saturation flow rate (SFR), as well as corresponding to the PCEs. Most of these works were observed to be carried out in lane-based and homogenous traffics. Furthermore, less existing work was found to comprehensively evaluate the PCEs and their effect on capacity, as well as level of pretimed signalized intersection services, within the heterogenous traffic.

These work efforts were more enhanced by proposing an automatic estimation method, based on video detector data [13] and dynamic evaluation of SFR [4]. Based on several studies, the probability method adopted by [14], was often used to model the effects of heterogeneous traffic on the SFR at a signalized intersection, by comparing the headway ratio and approaches. They further concluded that this method was more appropriate to rationalize the heterogeneous traffic in India. This was also observed in the heterogenous traffic within Bangladesh by [15], where a microscopic simulation approach was applied to model the passenger car equivalent (PCEs), through the considerations of the lane width characteristics and turning percentages of heavy and non-motorized vehicles.

Further studies showed that the capacity at a signalized intersection was substantially affected by the presence of non-motorized traffic and rickshaws, in the city of Dhaka, Bangladesh [16]. It was also concluded that the capacity decreased in the presence of non-motorized traffic and rickshaws. The recommendations stated that the adjustment of PCEs for non-motorized traffic and rickshaws should often be carried out, in order to effectively predict signalized capacity. Further investigations confirmed that the SFR was strongly influenced by the percentage of two-wheelers. Therefore, the higher percentage of the two-wheelers produced lower capacity. Additionally, the regression approach was used in modelling the PCE of two-wheelers [17].

The motorcycle homogenous traffic was further used to analyze flows, which were dominated by two-wheelers at signalized intersections in Hanoi, Vietnam. Furthermore, the motorcycle unit (MCU) was used to measure the SFR at a signalized intersection. A linear regression approach was also implemented to model the BSFR at a signalized intersection, while observing that the value of the two-wheeler unit for a car was 3.67 MCU [18]. Further calibration of the HCM (2000) was carried out by using observed data from three signalized intersections, in the city of Bangalore, India [19]. This showed that the high percentage of two-wheelers produced a large deviation in the SFR between the observed and estimated values, when using the HCM (2000). Additionally, the two-wheeler and heavy vehicle adjustment factors were also introduced and tested. Based on the results, it was concluded that the HCM (2000) should be used after considering the adjustment factors and PCEs, in order to use specific unit of PCU and SFR for highly heterogeneous traffic at urban signalized intersections [20].

Previous studies also showed that the adoption of HCM traffic manual required a calibration, by using local adjustment factors such as the PCEs. Conducting studies to model theories through locally observed data, was also found to be a prerequisite in the calibration of IHCM. Furthermore, this study is an initial contribution that provided a basic empirical study, in order to upgrade the existing IHCM PCEs to highly heterogeneous current traffic, especially at signalized intersections.

### Proposed model estimation

The linear regression is widely used to model and calibrate both PCE and SFR, as stated in section 2.1. This PCE was formulated through multiple linear regression, based on the assumption that an experiment consisting of exogenous variables, such as Y = {Y_i_}, had different values of independence X. Furthermore, an experiment with a stochastic nature was reported to have different values of Y_i_ for the same rate of X_i_. Assuming the probability distributions [*f*_*i*_ (Y|X)] had the same variance (σ2) for all values of X, a straight line known as the *true regression* was likely to be formed with the mean values of *μ*_*i*_ = *E* (*Y*_*i*_), as specified in Eq 3. Additionally, the population parameters (α and β) used to define the line, were estimated from the observed data set, in this equation.


$$\text{E}({\text{Y}}_{\text{i}})=\alpha +\beta {\text{X}}_{\text{i}}$$ (3)

The coefficient of parameters of α and β were calibrated using the dataset observed from the survey. One common method of achieving this process was through Ordinary Least Square (OLS). Furthermore, the Bayesian inference and regression parameter in the OLS were estimated by assuming and following certain prior distribution. This distribution (πθ) reflected the prior knowledge of the data set to be analyzed. The likelihood function was further used to update the prior distributions, which helped in obtaining the posteriority (π(θ|y)) of the parameters. Based on the assumption that represented parameters to be determined, the posterior distribution of the θ is formulated as follow,

$$\theta (\theta |\text{y})=\frac{f(\theta |\text{y})\pi (\theta )}{\int_{\theta }^{}(y|\theta )\pi (\theta )d\theta }\propto f(y|\theta )\pi (\theta )$$ (4)

where,

y = {y_1_,…,y_2_,…y_n_} represented the observed outcomes, π(y|θ) denoted the sampling distribution, $\int_{\theta }^{}(\text{y}|\theta )\pi (\theta )d\theta$ represents marginal distribution of y. The Bayesian inference provided a flexible framework, in order to integrate prior knowledge. It was also important for data with small sample sizes, which do not adequately represent the population [21,22]. This inference was performed by using both Random-walk Metropolis-Hastings and Gibbs sampling. Furthermore, the model convergence was monitored by the ratio of Monte Carlo errors, which were relative to the respective standard deviation of the estimates, at < 0.05 [22].

The PCU is known as a unit used to convert heterogeneous traffic into homogenous types, in terms of PCEs. Saturation time during green displays was presently regressed to total traffic compositions, which were observed during saturated time (green effective). Furthermore, the parameters should be clearly defined, in order to ensure saturated flow. The saturation time was found to be the period observed during maximum discharge flow, which should be maintained during the beginning of green effective. According to the on-site observation, the initial lost time (start-lag) was in the range of 4–5 secs, compared to the end-lag (additional time after yellow onset) at approximately 3 secs. It was also noted that the field observation showed continuous discharge flows during the end-lag, until the occurrence of the red phase. Therefore, the saturation time was presumed to be the sum of the effective green and end-lag times, in this study. The observed vehicle composition represented heterogeneous traffic, which comprises of motorcycles (MC), passenger cars (PC), motorized rickshaws (MR), and medium trucks/buses (TR/BS). Accordingly, the formulation of linear regression was expressed as,

$$\begin{array}{l} \text{T}=\alpha_{0}+\alpha_{\text{MC}}{\text{N}}_{\text{MC}}+\alpha_{\text{PC}}{\text{N}}_{\text{PC}}\\ +\alpha_{\text{RS}}{\text{N}}_{\text{MR}}+\alpha_{\text{BS}/\text{TR}}{\text{N}}_{\text{BS}/\text{TR}} \end{array}$$ (5)

The PCE was further calculated using Eq (6).

$${\text{PCE}}_{\text{i}}=\frac{\alpha_{\text{i}}}{\alpha_{\text{PC}}}$$ (6)

where,

T = the observed saturated time (s),
N = the observed number of vehicles discharged during the saturated time for MC, PC, MR, BS/TR,
α_0_ = start lost time,
α = headway corresponding to the type of vehicles of motorcycle (MC), passenger car (PC), motorized rickshaw (MR), medium bus and truck (BS/TR).

### Area of study and data collection

**Fig 1:**
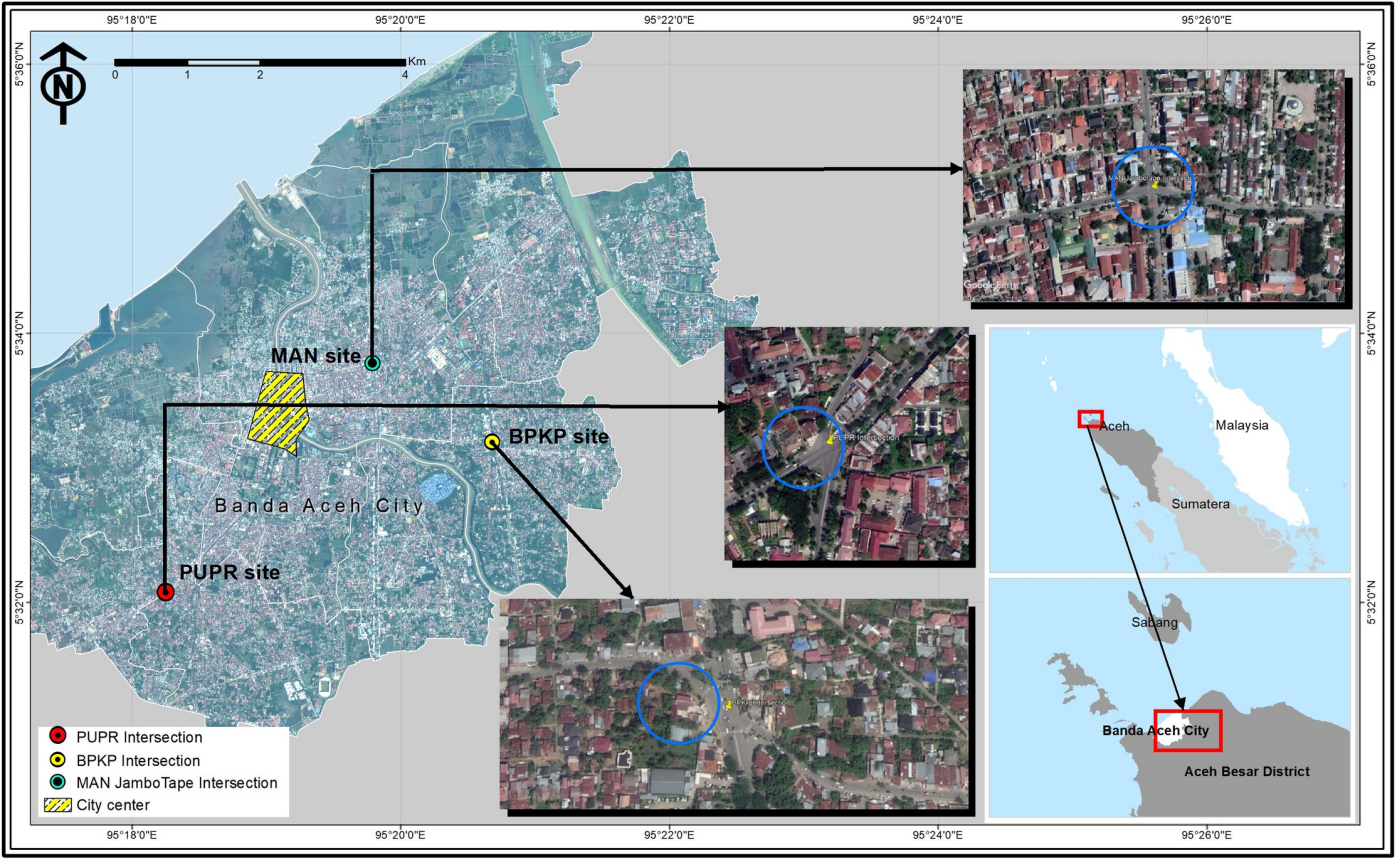
Map of study area (within city center of Banda Aceh, Aceh province).

Based on Fig 1, the target locations where the observed traffic data were collected was indicated. This included three pretimed signalized intersections located in Banda Aceh city, as well as various geometrical characteristics and vehicle compositions. The signal timing also had significant impact on the performance analysis of the signalized intersection [23]. Furthermore, four-legged pre-timed signalized intersections were considered as the target traffic facilities, namely MAN Jambo Tape, PUPR, and BPKP. These intersections were selected due to moderate traffic volumes, and approach widths in the range of 2.8–8 m. Also, the MAN Jambo Tape had all approaches that have left-turn canalization. Several previous studies also showed that turning movements and canalization substantially had effects on the determination of signalized intersection safety and operation [24,25]. Furthermore, the PUPR only had a northbound approach with a left-turn channelization. This intersection also had unsymmetric geometric features for north and southbound approaches, respectively. The BPKP also had similar geometric conditions with the PUPR, based on the west and eastbound approaches having left turn on red canalization, as illustrated in Fig 1. These three intersections were operated based on the conventional stage-based signal control [26], as each of the approaches had respective phase time.

According to this study, analysis was conducted based on the assumption that vehicular movements/trajectories within the targeted intersection were simplified. The modeling vehicle movement at a signalized intersection was also comprehensively investigated, by using several advanced approaches such as the extended car-following model [27]. Furthermore, the trajectories were modeled feature of microscopic intersectional vehicular movement, which was carried out by using a two-dimensional approach [28].

Based on the collection of data, geometric conditions such as the effective approach and lane widths, stop line position, dimensions of the intersection (including conflict area), and presence of left turn on red (LTOR), were initially measured directly on site. The traffic stream data, such as the numbers of straight movements, right and left turns (when LTOR is not permitted), as well as the type of vehicle for each movement, were observed directly on site, during the morning (07:15–08:50) and evening peaks (16:30–18:00). Conclusively, the signal timing data, such as green, red, and yellow intervals, as well as start and end-lag time, were directly measured on site, with correspondence to the morning and evening peak observations.

Geometric data were also obtained by surveyors, through direct measurement at the target intersections. The observation was conducted during off-peak hour traffic, in order to avoid difficulties due to vehicle movements. These data were recorded by filling survey forms, including the layout design of the intersections. Furthermore, the traffic data were recorded using a video camera for each approach. These cameras were placed on a tripod or mounted on a high-rise building, at the location of the survey. A total of 520 cycles of data were also extracted for each approach (40 cycles) of intersections, during the morning and evening observations. The signal timing further included start and end-lag times, which were directly measured on-site through a stopwatch. These time intervals were immediately recorded after the appearance of the green phase, until the driver in the front queue reacted. This condition assumed that 2–3 cars or 1–2 two-wheelers had been discharged from the stop line.

## Results and discussion

### Vehicle composition and PCE values

**Fig 2:**
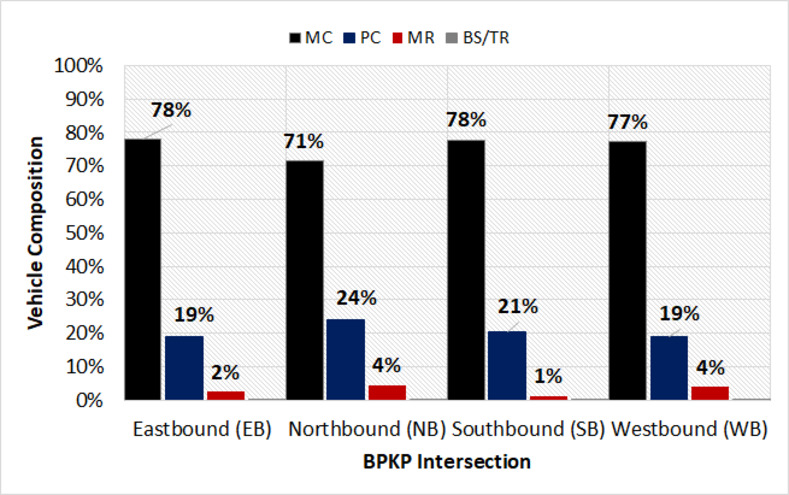
Vehicle composition at BPKP intersection.

**Fig 3:**
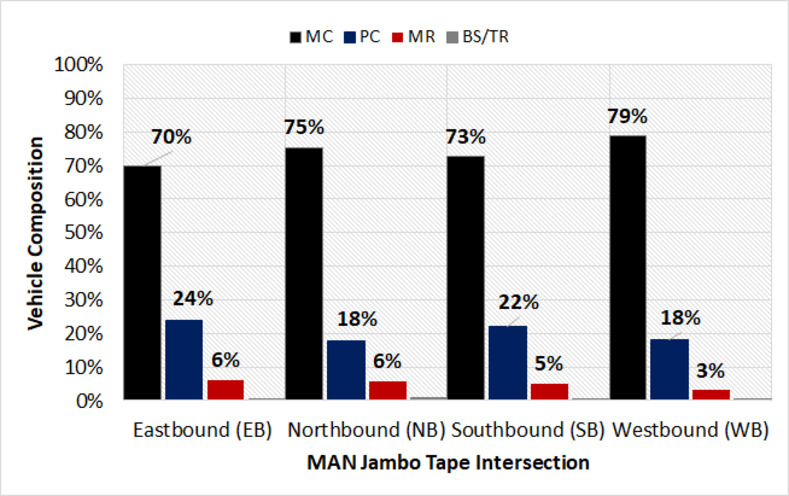
Vehicle composition at MAN Jambo Tape intersection.

**Fig 4:**
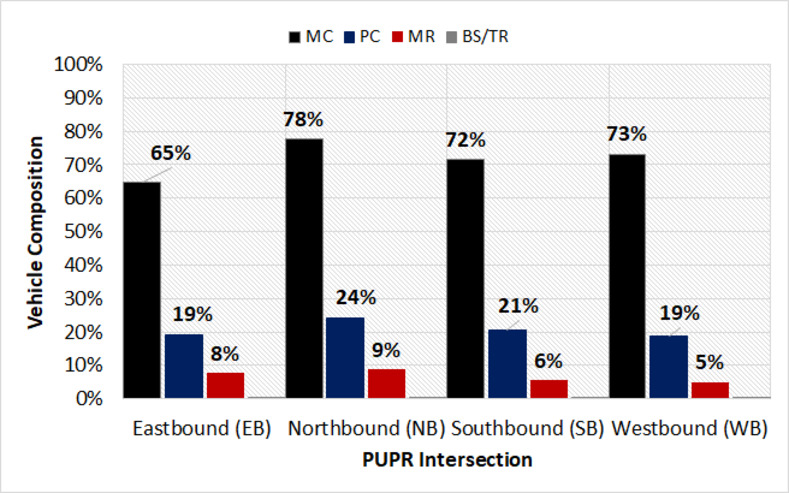
Vehicle composition at PUPR intersection.

Based on Figs 2–4, the traffic composition at the target signalized intersections was indicated. The tendencies of these compositions also considerably seemed the same for all the approaches. Furthermore, the most dominant type of traffic was motorcycles (MC), which were approximately 74% on average, accompanied by passenger cars (PC) at 21%. The remaining 5% of the traffic present was observed for motorized rickshaws (MR) and buses/trucks (BS/TR). It was also noted that the heavy vehicles (bus and truck) were restricted from entering the city center, during the peak hour traffic. Therefore, the BS/TR in this study was excluded from the analysis.

According to the use of 520 cycles as dataset, the formulation of linear regression was employed, in order to examine the headway of each vehicle types (i.e., MC, PC and MR). Based on the Bayesian inference, linear regression was conducted by using Random-walk Metropolis-Hastings sampling (MCMC iterations = 20,000; Burn-in = 10,000; MCMC sample size = 10,000). The result of this inference was also confirmed by using the Gibbs sampling (MCMC iterations = 12,500; Burn-in = 2,500; MCMC sample size = 10,000). Furthermore, the posterior estimates of the two model were found to be statistically similar. The Monte Carlo Standard Error (MSCE) was also less than 0.05, which was used to check the model convergency and significant estimated parameters. Based on these conditions, the summary of the final estimated model and its Goodness of fit are illustrated in Table 1.

**Table 1:** Model convergence and estimated parameter.

Variable	Bayesian linear regression (Random-walk Metropolis-Hastings’s sampling)				Bayesian linear regression (Gibbs’s sampling)			
	Mean	SD	MSCE	T-Stat	Mean	SD	MSCE	T-Stat
MC	0.137*	0.013	0.001	10.29	0.136*	0.013	0.000	10.743
PC	0.562*	0.044	0.002	12.90	0.562*	0.042	0.000	13.289
MR	0.446*	0.092	0.003	4.87	0.449*	0.095	0.001	4.705
CONS	9.545*	0.292	0.012	32.64	9.564*	0.296	0.003	32.341
SIGMA	9.109	0.571	0.012	15.96	9.106	0.570	0.006	15.969
***Goodness of Fit Model***								
N	520				520			
DIC	2,628.39				2,628.49			
LML	- 1,337.79				- 1,346.45			

Note: SD (Standard Deviation); MSCE (Monte Carlo Standard Error); T-Stat (T-Statistic = mean/SD); DIC (Deviance Information Criterion); LML (Log Marginal Likelihood);

*significant error at 1% level.

Table 1 depicted the estimated parameters and goodness of fit model for both proposed estimations. The explanatory variable of vehicles compositions, namely MC, PC and MR, were observed to be significantly affecting the saturation time of the signalized intersections. According to Eq (5), the CONS was represented by base headway, as the mean of parameters corresponding to the MC, PC and MR, was that (headway) of each vehicle types. The PCE was further determined through Eq (6), as the ratio of subjective vehicle to passenger car (PC) headways.

The volume unit of the vehicle/hour/lane was further found to be inappropriate under mixed conditions, as the traffic operated without lane discipline in Indonesia. Furthermore, the composition of vehicles through this mixed traffic was often diverse, consisting of light and heavy cars, motorcycles, rickshaws, etc. Each vehicle type was also found to have different effects on traffic, as a vehicular conversion factor was needed. The unit of this factor is known as passenger car unit (PCU). Additionally, the PCE value was considered as the critical part of capacity estimation. This was due to the capacity being often expressed as PCU/h, while the PCU was only determined through the PCEs [10].

Table 1 indicated that the log marginal likelihood and DIC among the two proposed models were not significantly different. This was due to the first (2,628.39) and second (2,628.49) models of the DIC substantially having similar values. However, the contrast in DIC among compared models that were less than 5, was considered to be insignificantly different [22].

The PCEs of each vehicle types were calculated through the ratio of their headways to the PC. According to the average headway, the result of the PCEs calculated were listed in Table 1. This illustration statistically indicated that headway parameters were assigned with significant estimated coefficients, as they showed greater T-Statistic value at 1% error level. Based on Table 1, the headway corresponding to BS/TR was excluded due to insignificant variables belonging to heavy vehicles, especially at the PUPR and MAN Jambo Tape locations. This was partially due to the low volumes of heavy trucks and buses, during peak hour observation at these locations. Therefore, the PCEs are listed in Table 2 below. Based on the average headway calculated in Table 1, the estimation of these PCEs were carried out through their mean values. This was due to the mean estimated parameters being significant (at 99% confidence interval), as the PCEs values for MC and MR were substantially acceptable. Based on Table 2, the obtained PCE value were found to be 0.24, 1.0, and 0.80 for motorcycle (MC), passenger car (PC), and motorized rickshaw (MR), respectively. Based on verifying the accuracy and rationality of estimated PCEs, a comparison approach was conducted with several values of the existing model, as shown in Table 2. The results showed that the values from the IHCM (existing PCEs) were substantially lower, compared to both proposed PCEs and existing literature [29–31]. It was also confirmed that the proposed PCEs significantly had similar values as those predicted from the existing model [29,30]. However, the proposed values [31] were found to have larger variations, compared to the PCEs in this study. These variations were attributed to the different proportions of vehicular composition and intersectional geometry, which were partially due to the turning behavior of vehicles. Additionally, the validation of the predicted PCEs (new PCEs) was also performed, through the comparisons with those from IHCM 1997.

**Table 2:** Obtained PCEs value.

Vehicle Type	New Proposed PCEs	Existing PCEs	PCEs from Literature (Estimated from other PCE models)		
MC	0.24	0.20^a^	0.25^b^	-	0.34^d^
PC	1.00	1.00	1.00	1.00	1.00
MR	0.80	0.50^a^	0.98^b^	0.86^c^	1.88^d^

Where, MC = motorcycle, PC = passenger car, MR = motorized rickshaw,

^a^ = Indonesian Highway Capacity Manual [9],

^b^ = Surbakti and Sembiring [29],

^c^ = Saha et al [30],

^d^ = Radhakrishnan and Mathew [31].

**Fig 5:**
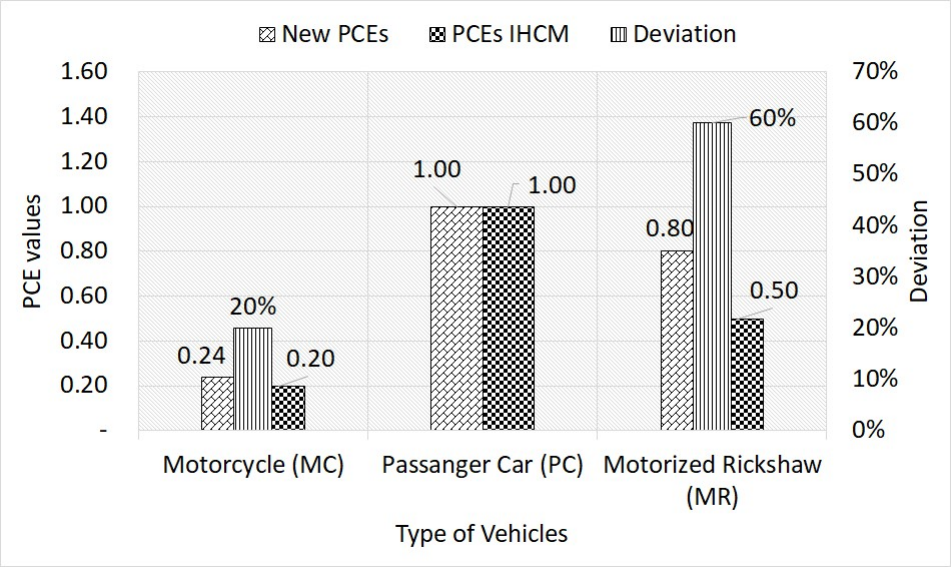
Validation result between new PCEs and PCEs from IHCM.

Fig 5 indicated the validation results between the new and IHCM PCEs, which showed that a larger deviation of 60% was found for motorized rickshaws (MR). This was probably due to the presently operated MR being motorize. The dimension of this present MR was also significantly larger than the conventional type, as Fig 5 was found to have illustrated the difference between motorized and non-motorized rickshaws (MR & NR), respectively. Furthermore, the deviation between both PCEs was due to the differences in the physical size (vehicle dimension), intersection geometry conditions, and vehicle compositions of the present type, compared to that of 1997, when the IHCM was published by the government. Also, the present performances of drivers were expected to be different, compared to the investigation of the IHCM. These significant variations substantially affected the prediction of the BSFR, based on the signalized intersections. The PCEs for MC also drastically altered the BSFR value, which was obtained through the IHCM formula. This was due to the traffic circumstances overflowing with more than 70% of two-wheelers. Therefore, it was concluded that the IHCM PCE value should be adjusted with the present prevailing conditions.

### Effect PCEs on predicting base saturation flow rate

The comparisons between efficiencies of IHCM and newly proposed PCEs were further carried out, in order to demonstrate their effects on the prediction of saturation flow rate (SFR), in the unit of PCU/h. According to Eqs 1 and 2, this saturation flow rate (S) was the multiplication between BSFR (S_0_ = 600We) and adjustment factors. The base saturation flow rate was also considered in order to demonstrate the effect of PCEs on the performance of pre-timed signal intersection. Firstly, the observed base saturation flow rate (Veh/h) was converted to PCU/h, through the IHCM 1997 and newly proposed PCEs. Secondly, the converted BSFR (y-axis) was found to be plotted against the effective width (We) on the horizontal axis. Furthermore, a simple linear trendline was used to perform data fitting, for both BSFR results obtained from IHCM and new proposed PCEs. The simple linear plot without intercept was selected, as it was found to be equal with the base saturation flow rate defined in IHCM (S_0_ = 600We). Conclusively, the linear plot of the new base saturation flow rate was compared and validated with the predictions of IHCM [9] and Munawar’s model [32].

**Fig 6:**
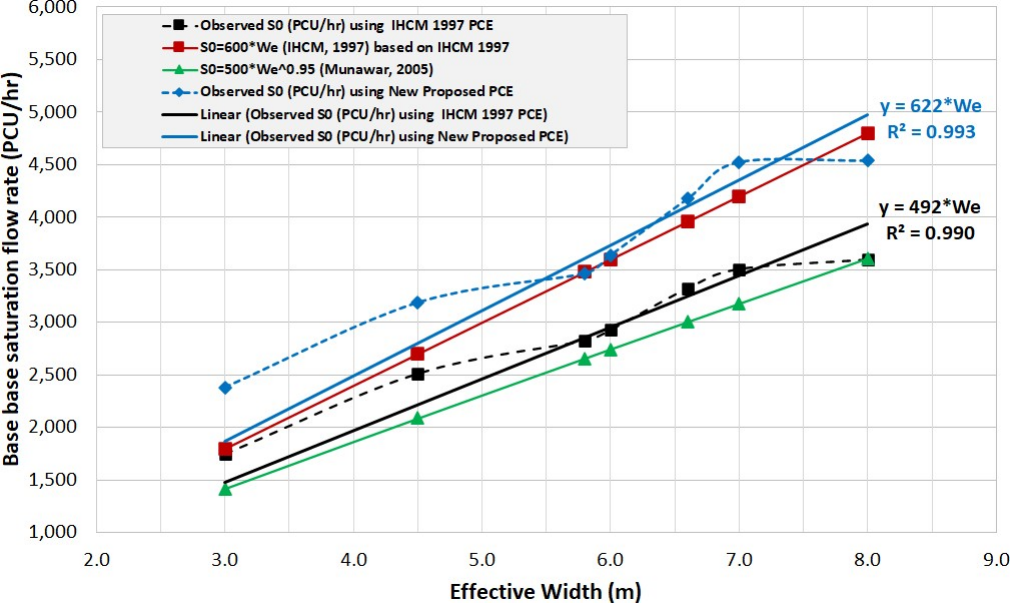
Comparing plot among base saturation flow rate model prediction using IHCM PCEs and new proposed PCEs.

Fig 6 illustrated the observed BSFR, after being converted into Passenger Car Unit (PCU), through the use of PCE. The observed BSFR generated from the IHCM PCEs (see black dotted line) was substantially similar to the predicted type, through the IHCM formula (red line). The result showed that valid estimation for approach widths was 3–4.5 m, with overestimate being observed to be larger than 5m. This prediction was substantially better than the BSFR estimated by Munawar’ model (green line). Furthermore, the BSFR predicted through the new proposed PCEs (blue line) was considerably more efficient (S_0_ = 622We), compared to that of the IHCM (S_0_ = 600We). It was also found that the observed data and newly adjusted model (S_0_ = 622We) indicated a valid estimation for effective widths, which was observed to be larger than 5m.

**Fig 7:**
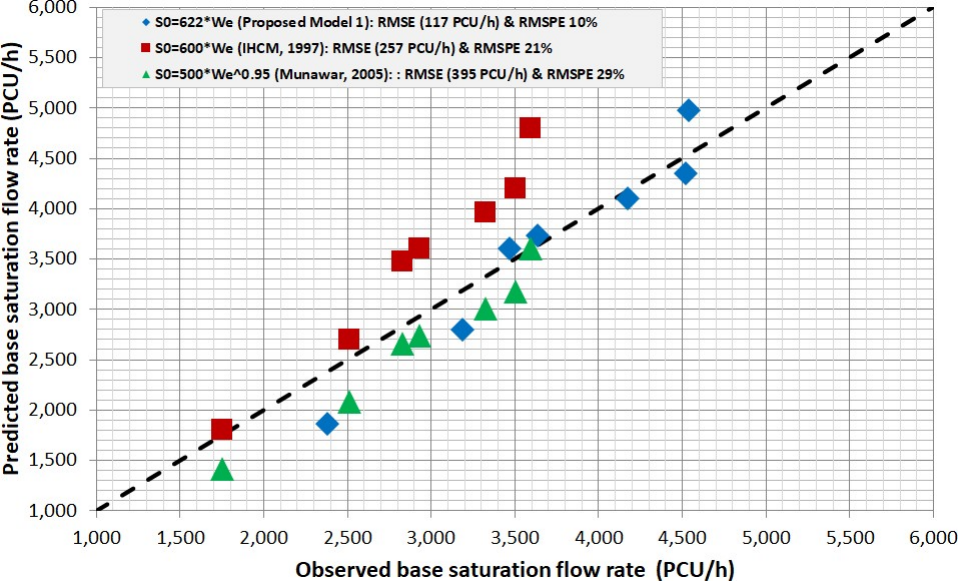
Validation plot among base saturation flow rate model prediction.

Fig 7 illustrated the visualization of a 45° line between the predicted and observed BSFR of three model, namely,

1. S_0_ = 600W_e_ [9],
2. S_0_ = 622W_e_ (Newly Proposed Model),
3. S_0_ = 500We^0.95^ [32].

The 45° line-marking showed that the predicted and observed BSFR data were randomly scattered. This verified that the newly proposed model substantially improved the prediction of BSFR, compared to both existing IHCM (S_0_ = 600W_e_) and Munawar’s (S_0_ = 500W_e_^0.95^) methods. Furthermore, the root mean standard and percentage errors (RMSE & RMSPE) were used as measures of effectiveness (MOEs), in order to ensure efficiency. The statistical measures of this errors also indicated that the IHCM model (S_0_ = 600W_e_) had higher RMSE of approximately 257 PCU/h (at RMSPE 21%), compared to the new proposed PCEs (S_0_ = 622W_e_) at 117 PCU/h (at RMSPE 10%). Conclusively, the BSFR from IHCM formula was observed to considerably need correction. This was due to avoiding the overestimation from unsuitable PCEs, which were within the present traffic composition and calibration parameter of BSFR at 600W_e_. Therefore, this result significantly affected the BSFR prediction, and led to the bias capacity estimation of pretimed signal intersection, especially for effective approach widths greater than 5m.

## Conclusion

The primary idea of this study was to determine new PCEs at pre-timed signalized intersections, considering the heterogeneous traffic and non-lane based movements. This study also presented the Bayesian inference, which was used in predicting the PCEs based on vehicle compositions and non-lane movements. Furthermore, the contributions were concluded as follows,

- The obtained PCE values were 0.24, 1.0, and 0.80 for motorcycle (MC), passenger car (PC), and motorized rickshaw (MR), respectively.
- A significant deviation was found between the newly proposed and IHCM PCEs. Also, a greater deviation was observed in the PCE motorized rickshaws (MR), at 56%. This was largely due to the differences in the physical size (vehicle dimension), drivers’ performance, and vehicle compositions.
- The BSFR predicted through the newly proposed PCEs was considerably more efficient (S_0_ = 622W_e_), compared with that of the IHCM (S_0_ = 600W_e_). Also, it was found that the IHCM prediction mode of BSFR seemed to be a valid estimation for effective widths less than 5m.
- The BSFR from the IHCM formula was observed to considerably need correction, this was due to avoiding the overestimation from unsuitable PCEs, which were within present traffic composition and calibration parameter of BSFR at 600We. This result significantly affected the BSFR prediction and led to the bias capacity estimation for effective approach widths greater than 5m.

The limitation of this study was that the data collected were only from the three busiest intersections in Banda Aceh, Aceh province, Indonesia. Therefore, this study should be extended by collecting more data from several cities in Aceh Province or even other locations within Indonesia. This should be carried out in order to enhance the validity dataset for calibrating the PCEs and BSFR of IHCM 1997, especially for pretimed signal timing intersection. Based on this study, the calibration of PCEs was proposed by considering only vehicular compositions at pretimed signalized intersections. The mechanism of vehicle movements inside intersections, geometric designs, and different signal control methods, also significantly affected the PCEs, and was further considered to be limited in the model hypothesis. This remained the future direction of the study, based on gaining more insight, enhancing the accuracy of base saturation flow prediction, and improving the estimation of signalized intersection capacity.

## References

[1] Saleh SM, Sugiarto S, Hilal A, Ariansyah D. A study on the traffic impact of the road corridors due to flyover construction at Surabaya intersection, Banda Aceh of Indonesia. AIP Conference Proceedings. 2017;1903: 060005. doi: 10.1063/1.5011559

[2] Sugiarto -, Limanoond T. Impact of On-street Parking on Urban Arterial Performance: A Quantitative Study on Travel Speed and Capacity Deterioration. Aceh International Journal of Science and Technology. 2013;2: 63–69. doi: 10.13170/aijst.2.2.697

[3] Sugiarto S, Limanoond T, Nakatsuji T. Dropped in Capacity and Traffic Speed of Urban Arterial: A Case Study at U-Turn Section in Aceh Province, Indonesia. Aceh International Journal of Science and Technology. 2012;1: 86–93. doi: 10.13170/aijst.1.3.136

[4] Wang Y, Rong J, Zhou C, Gao Y. Dynamic Estimation of Saturation Flow Rate at Information-Rich Signalized Intersections. Information. 2020;11: 178. doi: 10.3390/info11040178

[5] Wang Y, Rong J, Zhou C, Chang X, Liu S. An Analysis of the Interactions between Adjustment Factors of Saturation Flow Rates at Signalized Intersections. Sustainability. 2020;12: 665. doi: 10.3390/su12020665

[6] Qin Z, Zhao J, Liang S, Yao J. Impact of Guideline Markings on Saturation Flow Rate at Signalized Intersections. In: Journal of Advanced Transportation. Hindawi; 8 Apr 2019 p. e1786373. 10.1155/2019/1786373.

[7] Xuan Y, Daganzo CF, Cassidy MJ. Increasing the capacity of signalized intersections with separate left turn phases. Transportation Research Part B: Methodological. 2011;45: 769–781. doi: 10.1016/j.trb.2011.02.009

[8] Yan C, Jiang H, Xie S. Capacity optimization of an isolated intersection under the phase swap sorting strategy. Transportation Research Part B: Methodological. 2014;60: 85–106. doi: 10.1016/j.trb.2013.12.001

[9] IHCM. Indonesian Highway Capacity Manual. Directorate General of Highways of Republic Indonesia; 1997.

[10] Sugiarto S, Apriandy F, Faisal R, Saleh SM. Measuring Passenger Car Unit (PCU) at Four Legged Roundabout using Time Occupancy Data Collected from Drone. Aceh Int J Sci Technol. 2018;7: 77–84. doi: 10.13170/aijst.7.2.8587

[11] Branston D, Gipps P. Some experience with a multiple linear regression method of estimating parameters of the traffic signal departure process. Transportation Research Part A: General. 1981;15: 445–458. doi: 10.1016/0191-2607(81)90112-6

[12] Branston D, van Zuylen H. The estimation of saturation flow, effective green time and passenger car equivalents at traffic signals by multiple linear regression. Transportation Research. 1978;12: 47–53. doi: 10.1016/0041-1647(78)90107-7

[13] Wang L, Wang Y, Bie Y. Automatic Estimation Method for Intersection Saturation Flow Rate Based on Video Detector Data. In: Journal of Advanced Transportation. Hindawi; 17 Jul 2018 p. e8353084. 10.1155/2018/8353084.

[14] Thamizh Arasan V, Jagadeesh K. Effect of Heterogeneity of Traffic on Delay at Signalized Intersections. J Transp Eng. 1995;121: 397–404. doi: 10.1061/(ASCE)0733-947X(1995)121:5(397)

[15] Hossain M. Estimation of saturation Flow at signalised intersections of developing cities: a micro-simulation modelling approach. 2001; 19.

[16] Rahman MdM, Okura I, Nakamura F. Effects of Rickshaws and Auto-Rickshaws on The Capacity of Urban Signalized Intersections. IATSS Research. 2004;28: 26–33. doi: 10.1016/S0386-1112(14)60089-3

[17] Chu M, Sano K. Analysis of motorcycle effects to saturation flow rate at signalized intersection in developing countries. Journal of the Eastern Asia Society for Transportation Studies. 2003;5: 120–128.

[18] Nguyen Q. Saturation flow and vehicle equivalence factors in traffic dominated by motorcyles. 2007.

[19] Anusha CS, Verma A, Kavitha G. Effects of Two-Wheelers on Saturation Flow at Signalized Intersections in Developing Countries. J Transp Eng. 2013;139: 448–457. doi: 10.1061/(ASCE)TE.1943-5436.0000519

[20] Radhakrishnan P, Mathew TV. Passenger car units and saturation flow models for highly heterogeneous traffic at urban signalised intersections. Transportmetrica. 2011;7: 141–162. doi: 10.1080/18128600903351001

[21] Xie Y, Zhang Y, Liang F. Crash Injury Severity Analysis Using Bayesian Ordered Probit Models. J Transp Eng. 2009;135: 18–25. doi: 10.1061/(ASCE)0733-947X(2009)135:1(18)

[22] Yuan Q, Xu X, Zhao J, Zeng Q. Investigation of injury severity in urban expressway crashes: A case study from Beijing. Chen F, editor. PLoS ONE. 2020;15: e0227869. doi: 10.1371/journal.pone.0227869 31929601 PMC6957292

[23] Bang K, Wahlstedt J, Linse L. Methodology for Timing and Impact Analysis of Signalized Intersections. Transportation Research Procedia. 2016;15: 75–86. doi: 10.1016/j.trpro.2016.06.007

[24] Al-Kaisy A, Roefaro S. Channelized right-turn lanes at signalized intersections: the U.S. experience. 2012; 13.

[25] Gowri A, Sivanandan R. EVALUATION OF LEFT TURN CHANNELIZATION AT A SIGNALIZED INTERSECTION UNDER HETEROGENEOUS TRAFFIC CONDITIONS. TRANSPORT. 2008;23: 221–229. doi: 10.3846/1648-4142.2008.23.221-229

[26] Wang F, Tang K, Li K, Liu Z, Zhu L. A Group-Based Signal Timing Optimization Model Considering Safety for Signalized Intersections with Mixed Traffic Flows. Journal of Advanced Transportation. 2019;2019: e2747569. doi: 10.1155/2019/2747569

[27] Zhao J, Li P. An extended car-following model with consideration of speed guidance at intersections. Physica A: Statistical Mechanics and its Applications. 2016;461: 1–8. doi: 10.1016/j.physa.2016.05.042

[28] Zhao J, Knoop VL, Wang M. Two-dimensional vehicular movement modelling at intersections based on optimal control. Transportation Research Part B: Methodological. 2020;138: 1–22. doi: 10.1016/j.trb.2020.04.001

[29] Surbakti MS, Sembiring I. Passenger car equivalents of becak bermotor at road segment in Medan. IOP Conf Ser: Mater Sci Eng. 2018;309: 012105. doi: 10.1088/1757-899X/309/1/012105

[30] Saha P, Mahmud HMI, Hossain QS, Islam MDZ. PASSENGER CAR EQUIVALENT (PCE) OF THROUGH VEHICLES AT SIGNALIZED INTERSECTIONS IN DHAKA METROPOLITAN CITY, BANGLADESH. IATSS Research. 2009;33: 99–104. doi: 10.1016/S0386-1112(14)60248-X

[31] Radhakrishnan P, Mathew TV. Passenger car units and saturation flow models for highly heterogeneous traffic at urban signalised intersections. Transportmetrica. 2011;7: 141–162. doi: 10.1080/18128600903351001

[32] Munawar A. Queues and Delays at Signalized Intersections, Indonesian Experience. 2006. Available: https://trid.trb.org/view/792900.

